# Indoleacetylglutamine Pathway Is a Potential Biomarker for Cardiovascular Diseases

**DOI:** 10.3390/biom15030377

**Published:** 2025-03-05

**Authors:** Khaled Naja, Najeha Anwardeen, Mashael Al-Shafai, Mohamed A. Elrayess

**Affiliations:** 1 Biomedical Research Center, QU Health, Qatar University, Doha P.O. Box 2713, Qatar; khaled.naja@qu.edu.qa (K.N.); n.anwardeen@qu.edu.qa (N.A.); malshafai@qu.edu.qa (M.A.-S.); 2 Department of Biomedical Sciences, College of Health Sciences, QU Health, Qatar University, Doha P.O. Box 2713, Qatar; 3 College of Medicine, QU Health, Qatar University, Doha P.O. Box 2713, Qatar

**Keywords:** cardiovascular diseases, metabolomics, indoleacetylglutamine, gut microbiota

## Abstract

Cardiovascular diseases (CVDs) remain a leading cause of global morbidity and mortality. Metabolomics allows for the identification of important biomarkers for CVDs, essential for early detection and risk assessment. This cross-sectional study aimed to identify novel metabolic biomarkers associated with CVDs using non-targeted metabolomics. We compared the metabolic profiles of 112 patients with confirmed CVDs diagnosis and 112 gender- and age-matched healthy controls from the Qatar Biobank database. Orthogonal partial least square discriminate analysis and linear models were used to analyze differences in the level of metabolites between the two groups. We report here a significant association between the indoleacetylglutamine pathway and cardiovascular diseases, expanding the repertoire of gut microbiota metabolites linked to CVDs. Our findings suggest that alterations in gut microbiota metabolism, potentially resulting in increased production of indoleacetate, indoleacetylglutamine, and related compounds at the expense of the cardioprotective indolepropionate, may contribute to this association. Our findings may pave the way for novel approaches in CVD risk assessment and potential therapeutic interventions targeting the gut-heart axis.

## 1. Introduction

Cardiovascular diseases continue to be a leading cause of morbidity and mortality globally, accounting for approximately 17.9 million deaths annually, which represents 32% of all global deaths [1,2]. Effective prevention strategies focus on identifying high-risk individuals well before significant cardiovascular events occur, emphasizing the importance of early detection and management [3]. In addition to traditional risk factors, extensive research has led to the identification of novel biomarkers that enhance risk stratification, allowing for better prediction of cardiovascular events and improved patient outcomes [4,5]. These biomarkers reflect various aspects of atherosclerosis development and include inflammatory markers, myocardial tissue-specific proteins, and other indicators that contribute to more accurate risk assessments and treatment decisions [5]. Metabolic markers are among the key biomarkers being explored for their predictive value in CVD risk assessment. These biomarkers include, but are not limited to, acylcarnitines [6,7], branched-chain amino acids [8], bile acids [9], and microbiota-derived metabolites [10]. The integration of various biomarkers from cardiac, metabolic, and other pathways holds great promise in improving the accuracy of CVD risk assessment and prognosis.

Metabolomics enables the identification of diagnostic and prognostic biomarkers for CVDs, which are crucial for early detection and risk stratification [11]. This capability is integral to the development of metabolomic profiles, which can assist in the diagnosis and management of CVDs [12]. Studies have identified specific metabolite patterns associated with various cardiovascular conditions, such as coronary artery disease, heart failure, and atrial fibrillation, which can potentially enhance risk assessment and guide preventive interventions [13,14,15]. Metabolomics can also identify metabolic biomarkers of gut microbiota, which are increasingly acknowledged to play a significant role in cardiometabolic health [11]. The integration of metabolomics into clinical practice has the potential to significantly impact public health by reducing the burden of CVDs. Although it is not yet widely used in clinical settings, it is possible to develop a metabolomic panel using patient serum to assess disease severity and predict disease progression. This panel would facilitate targeted and precision treatment approaches.

In this cross-sectional study, we conducted non-targeted metabolomics on serum samples to compare the metabolic profiles of 112 patients with cardiovascular diseases and 112 gender- and age-matched healthy controls. Our results revealed a significant alteration in the indoleacetylglutamine pathway associated with CVDs. We observed increased levels of indoleacetylglutamine, its precursor indoleacetate (indole-3-acetic acid), and its metabolite methyl indole-3-acetate, along with decreased levels of the cardioprotective indolepropionate in CVD patients. This finding suggests a potential metabolic shift in tryptophan metabolism by gut microbiota, further highlighting the intricate relationship between gut microbial metabolites and cardiovascular health.

## 2. Materials and Methods

### 2.1. Data Source and Study Participants

This study gathered data from Qatar Biobank (QBB). The QBB database contains detailed information about Qatari nationals and long-term residents (those living in Qatar for 15 or more years) who are 18 years old and above. It includes basic personal details, health information such as body mass index, blood pressure, and blood test results, as well as information about CVDs history, medications, and metabolomics data on 1000 different metabolites [16]. All these measurements were performed at the Hamad Medical Corporation’s central laboratory, which is certified by the College of American Pathologists. This research was approved by the Qatar Biobank’s institutional review boards (QF-QBB-RES-ACC-00178).

Six-hundred twenty-three participants in this study were divided into two groups: controls and individuals with CVDs. The inclusion criteria for the CVD group (*n* = 173) required participants to have a documented self-reported diagnosis of CVDs (e.g., who reported a history of heart attack, coronary artery disease, angina, or stroke) and to be actively receiving treatment with at least one medication aimed at managing their condition as verified by prescription records. The control group (*n* = 450) included participants who had no history of CVDs and were not diagnosed with any cardiovascular conditions. Individuals in both groups were excluded if they had comorbidities that could interfere with the results, such as severe renal insufficiency, chronic obstructive pulmonary disease, or cancer. Additionally, pregnant or breastfeeding women were excluded from both groups. Using propensity score matching, the age, BMI, and gender were matched using the R (matchit) package (V. 4.2.1), resulting in a total of 224 analyzed participants with *n* = 112 in each group. This will help to reduce confounding and increase the validity of the study’s findings.

### 2.2. Metabolomics

All participant serum samples were subjected to untargeted metabolomics using established protocols by Metabolon [17]. Metabolites measurement was performed using a Thermo Scientific Q-Exactive high resolution/accurate mass spectrometer (Thermo Fisher Scientific, Inc., Waltham, MA, USA) interfaced with a heated electrospray ionization (HESI-II) source and Orbitrap mass analyzer operated at 35,000 mass resolution along with Waters ACQUITY ultra-performance liquid chromatography (UPLC) (Waters Corporation, Milford, MA, USA). A thorough explanation of the process has already been provided [17]. In brief, the serum samples were initially processed through methanol extraction to remove proteins. The extracted samples were then divided into five parts: two parts were analyzed with different reverse-phase UPLC-MS/MS techniques with positive ion mode electrospray ionization (ESI), one part was analyzed with reverse-phase UPLC-MS/MS with negative ion mode ESI, another part was analyzed with hydrophilic interaction chromatography (HILIC)-UPLC-MS/MS with negative ion mode ESI, and the final part was reserved as a backup sample.

Hits were matched with pre-existing library entries of over 3300 pure standard chemicals to identify the compounds. Compounds were divided into several groups according to their sources. Internal standards and quality checks have been previously published [18]. In short, to adjust for discrepancies in sample preparation and instrument performance, a combination of stable isotope-labeled chemicals was utilized as internal standards. The stability and repeatability of the procedure were tracked over time using quality control samples. To reduce variability and guarantee the integrity of the samples, a systematic methodology was employed for pre-analytical sample management, including sample collection, storage, and preparation.

### 2.3. Statistical Analysis

The clinical data (anthropometrics, blood pressure, lipid, liver, and kidney profiles) were compared between the two groups using Student’s *t* test/Mann–Whitney U test based on their Gaussian distribution test results. The metabolomics data were log-normalized. SIMCA^®^ was used to perform multivariate analyses: Principal Component Analysis for quality control and orthogonal partial least squares discriminant analysis to identify metabolites associated with each group. R language (V. 4.2.1) was used to fit linear models using the in-built lm (y~x) function: the primary independent variable (x) in linear regression analysis was the control/CVD classification, whereas the dependent variable (y) was each metabolite. The model included the first two principal components from PCA analysis, age, gender, and body mass index (BMI) as confounding variables. False Discovery Rate (FDR) was used to adjust nominal *p*-values, and FDR < 0.05 was considered significant. Spearman’s correlation was performed between the top FDR-significant metabolites and clinical parameters in the cohort. To assess the discriminatory potential of indoleacetylglutamine and other candidate metabolites for CVD, receiver operating characteristic (ROC) curve analysis was performed.

## 3. Results

### 3.1. General Characteristics of Participants

We employed propensity score matching techniques to balance the demographic characteristics of age, BMI, and gender between groups. As a result of this procedure, the final sample consisted of 224 participants, evenly distributed with 112 individuals in each group. Despite the application of propensity matching to balance age, gender, and BMI between groups, Table 1 shows that age and BMI remained significantly different between the study groups. However, the difference was notably reduced, compared to the unmatched dataset.

### 3.2. Multivariate Analysis

**Figure 1:**
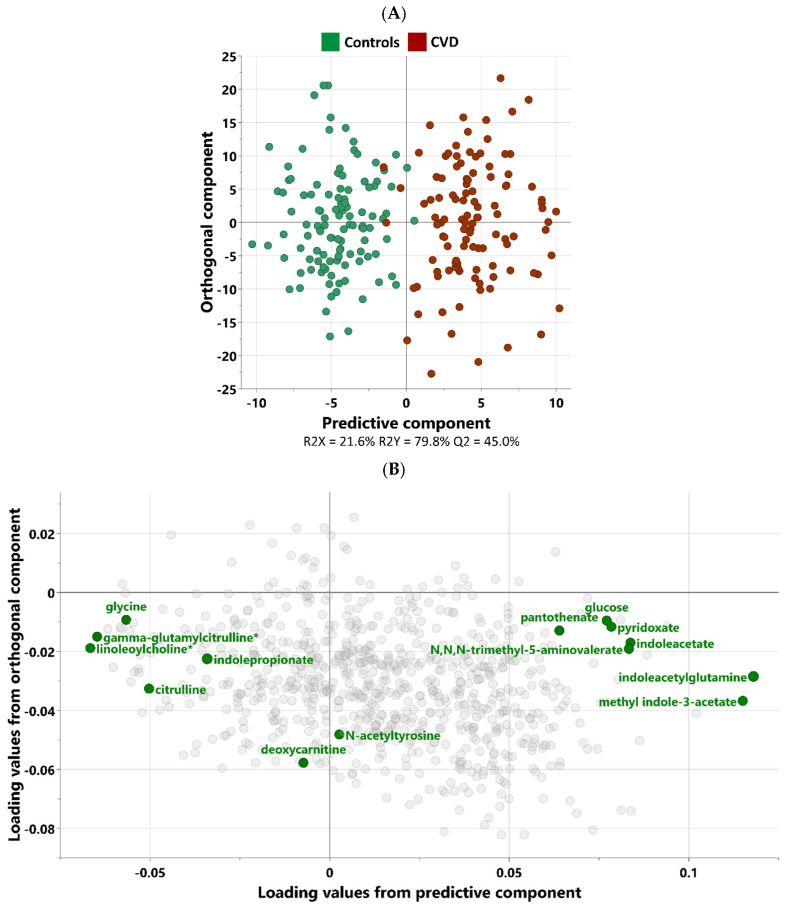
OPLS-DA score (**A**) and loading plots (**B**), depicting the metabolic profile difference and most discrepant metabolites between CVD (*n* = 112) and control (*n* = 112) individuals. OPLS-DA illustrates the clear separation between CVD patients and healthy control individuals based on their metabolic profiles. Each point represents an individual subject. The model identified one predictive and three orthogonal components (R2Y = 0.798; Q2 = 0.450). The loading plot reveals the metabolites most responsible for the separation between CVD and control groups. Metabolites highlighted in green are the key discriminators, while less influential metabolites are shown in gray to reduce visual noise. * indicates a compound that has not been officially confirmed based on a standard, but that Metabolon is confident in its identity.

Non-targeted metabolomics analysis was performed to investigate the metabolic signatures of the CVD group compared to the control group. Orthogonal partial least squares discriminant analysis (OPLS-DA) was used to identify the best distinguishing components between the two groups as shown in Figure 1. The scatter plot in Figure 1A clearly exhibits the distinct separation of the two groups. Figure 1B displays the corresponding loading plots, revealing the primary metabolites responsible for distinguishing the two groups. These include indole-related metabolites, glucose, N-acetyltyrosine, deoxycarnitine, and N-acetyltyrosine.

### 3.3. Univariate Analysis

**Figure 2:**
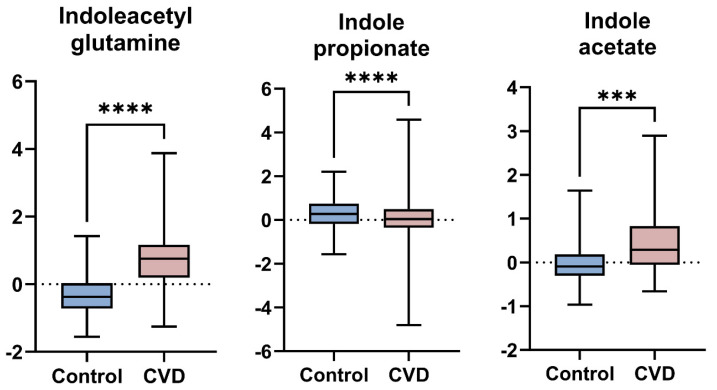
Boxplots comparing metabolite levels between CVD and control groups. *** FDR < 0.001, **** FDR < 0.0001.

Linear model analysis revealed a number of FDR (≤0.05) significant changes between the two studied groups. Table 2 and Figure 2 show the most significant metabolites. Results show an increase in indoleacetylglutamine, methyl indole-3-acetate, and indoleacetate, and a decrease in indolepropionate in the CVD group. The full list of metabolites is provided in Supplementary Table S1.

### 3.4. Spearman’s Correlation Analysis

**Figure 3:**
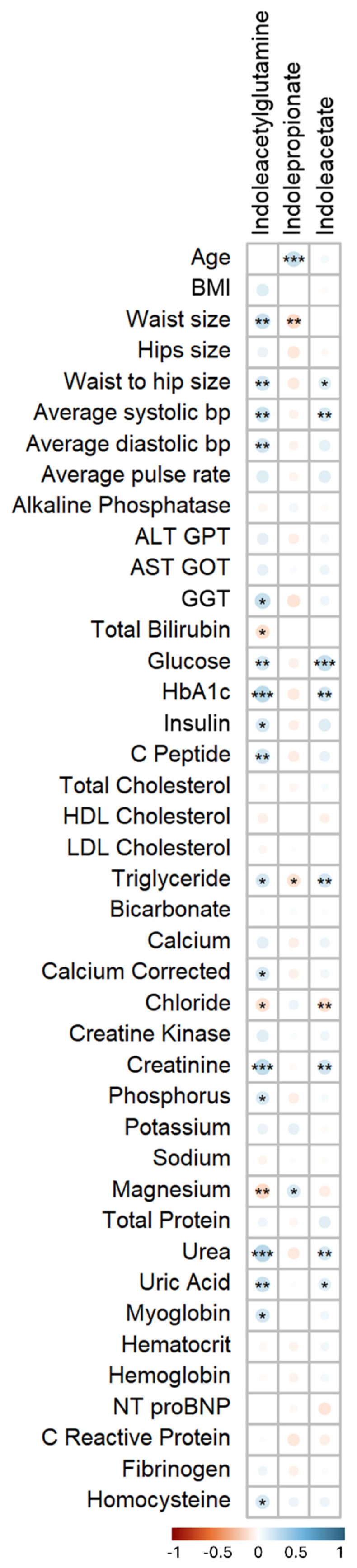
Heatmap of correlation between the FDR-significant metabolites and clinical traits performed using Spearman’s correlation test. The size of the circles within each cell corresponds to the magnitude of Spearman’s correlation coefficient. The color intensity in each cell represents the strength and direction of the correlation between a specific metabolite and a clinical trait. (***/**/*) signifies *p*-value < 0.001/<0.01/<0.05, respectively.

Spearman’s correlation analysis (Figure 3) revealed two distinct patterns among the top FDR metabolites in our study. Indoleacetylglutamine and indoleacetate show significant positive correlations with BMI, waist size, and hip size, indicating a link to obesity-related metrics. They also correlate positively with triglycerides, a key marker of dyslipidemia, which is a major risk factor for CVD. Additionally, indoleacetylglutamine is positively associated with uric acid, which has been implicated in hypertension and endothelial dysfunction, factors that elevate CVD risk. In contrast, indolepropionate exhibits a reverse pattern and appears to be cardioprotective. It shows a significant positive correlation with magnesium, which is known to reduce arterial stiffness, improve endothelial function, and lower blood pressure.

## 4. Discussion

Early detection and risk stratification using novel biomarkers and metabolomics are essential for reducing the global impact of cardiovascular diseases and improving public health strategies. Our study identifies indoleacetylglutamine pathway as a significant metabolic pathway associated with cardiovascular diseases. Thus, providing insights into its potential role in the pathophysiology of CVDs, particularly through gut microbiota-derived metabolic pathways.

Our multivariate and univariate analysis show a significant elevation of indoleacetylglutamine in the CVDs group. Additionally, its precursor, indoleacetate (also known as indole-3-acetic acid), and one of its metabolites, methyl indole-3-acetate, were also found to be significantly increased in this group. These findings suggest a potential metabolic pathway that may be associated with the pathophysiology of CVDs. Indole-3-acetic acid (IAA) is an important indole metabolite metabolized from tryptophan fermentation by the gut microbiome [19]. In the liver, IAA can combine with glutamine to produce indoleacetylglutamine [20]. Dou et al. [21] reported that high IAA is associated with increased cardiovascular events and mortality in patients with chronic kidney disease (CKD), primarily through mechanisms of endothelial inflammation and oxidative stress. Subsequent studies [22,23] have consistently supported these findings, highlighting a significant association between IAA and CVDs, particularly in the context of CKD. Data on indole-3-methyl acetate are very limited. Stoll et al. [24] reported altered indole-3-methyl acetate levels among several tryptophan metabolites in pediatric enthesitis-related arthritis fecal samples.

Recent studies have highlighted the crucial role of gut microbiota-derived metabolites in human health, particularly in relation to cardiovascular outcomes [25,26]. Gut microbiota dysbiosis frequently occurs before the onset of clinical symptoms, implying that gut microbial imbalance could be a primary trigger for cardiovascular diseases [27]. Moreover, the use of probiotics, to modulate the gut microbiota, has demonstrated protective effects against cardiovascular disease, highlighting the importance of gut microbiota in maintaining cardiovascular well-being [28]. For instance, the association between phenylacetylglutamine and CVDs is well established [29], with several studies confirming its link to heart failure, coronary heart disease, and other cardiovascular conditions [30,31,32,33,34]. The mechanisms underlying these associations are thought to involve phenylacetylglutamine effects on adrenergic receptor signaling, platelet activation, and endothelial dysfunction [30]. However, emerging evidence suggests that other aromatic amino acids-derived metabolites may also play significant roles in cardiovascular health [35].

Lee et al. [36] notably reported that both phenylacetylglutamine and indoleacetylglutamine, in addition to trimethylamine N-oxide, are linked to negative neurocognitive outcomes in pediatric CKD patients. Moreover, Nemet et al. [37] contributed to an atlas mapping the relationships between gut microbial metabolites and cardiovascular risks, revealing that phenylacetylglutamine and indoleacetylglutamine are associated with a higher incidence of major adverse cardiovascular events.

Our results are further validated by the significantly decreased levels of indolepropionate in the CVDs group. Indeed, indolepropionate (or indole-3-propionic acid), which is also a gut microbiota-derived tryptophan metabolite, has shown significant cardioprotective effects in various CVDs models. Indolepropionate improves mitochondrial function in cardiomyocytes [38], reduces atherosclerosis progression [39], and protects against diastolic dysfunction in heart failure with preserved ejection fraction [40].

Our correlation analysis further corroborates these findings by revealing distinct cardiovascular and metabolic risk patterns associated with the studied metabolites. Indoleacetylglutamine and indoleacetate demonstrate significant positive correlations with blood and key metabolic parameters, including glucose, HbA1c, insulin, and C-peptide. These associations suggest their potential involvement in pathways linked to impaired glucose regulation and hypertension. Notably, indoleacetylglutamine shows strong positive correlations with waist size and waist-to-hip ratio, highlighting its connection to central adiposity and potential contributions to obesity-related cardiovascular risk. In contrast, indolepropionate exhibits a markedly different profile, characterized by negative correlations with BMI, waist size, waist-to-hip ratio, triglycerides, and positive correlations with HDL cholesterol and magnesium. These findings align with its potential protective role in cardiovascular health by promoting a favorable lipid profile and reducing markers of obesity.

To assess the predictive ability of indoleacetylglutamine and other metabolites for CVDs, we performed a receiver operating characteristic (ROC) curve analysis. The ROC analysis (Supplementary Figure S1) demonstrated that these metabolites effectively differentiate individuals with and without CVDs, thereby highlighting their potential as predictive biomarkers.

**Figure 4:**
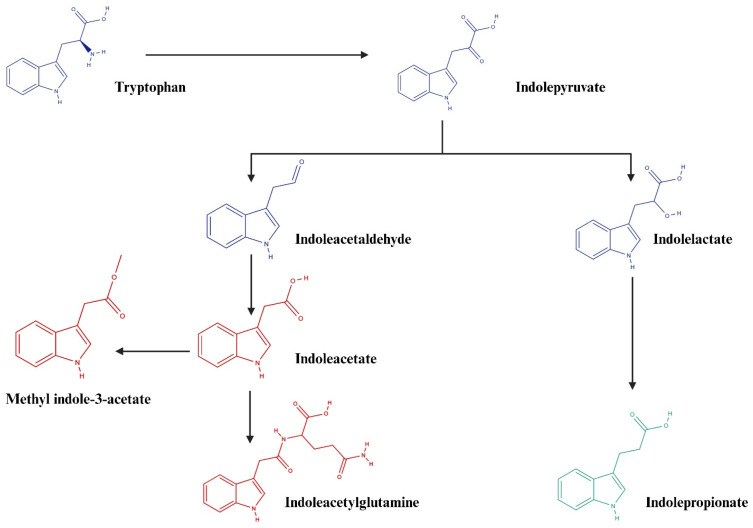
Metabolic pathways significantly associated with CVDs in this study. Red and green colors, respectively, represent metabolites that are elevated and decreased in CVDs compared to healthy controls. Created in BioRender (www.biorender.com).

Several microbes producing tryptophan catabolites have been identified [19], but no studies have yet used human data to pinpoint the main producers in the gut. In our study, the concomitant increase in indoleacetylglutamine and indoleacetate and the decrease in indolepropionate in the CVD group could be explained by a dysbiosis in the gut microbiota which divert their metabolism into a pathway that favors the production of indoleacetate and related metabolites at the expenses of indolepropionate (See Figure 4). These findings suggest that manipulating gut microbiota may be a therapeutic possibility for the treatment of many diseases associated with tryptophan metabolism.

The study presents several limitations that should be acknowledged. The cross-sectional nature of the study limits the ability to establish causal relationships between identified metabolites and CVDs. Furthermore, while this study identifies potential associations, it cannot definitively conclude the predictive ability of these metabolites for future cardiovascular events. Additionally, it should be considered preliminary as the sample size may still be insufficient to capture the full spectrum of metabolic variability across different populations. It is important to note that these metabolites may not improve CVD risk assessment beyond that of classic risk factors; however, our findings hold promise for an improved understanding of the pathophysiology of CVDs and will help focus future studies on gut–microbial metabolic outputs relevant to host cardiovascular health. Future studies include conducting large-scale, prospective studies to track microbiome changes across the cardiovascular risk spectrum and during acute events.

## 5. Conclusions

In this study, we identified indoleacetylglutamine as a significant metabolite associated with cardiovascular diseases, providing new insights into their pathophysiology through gut microbiota-derived metabolic pathways, and reinforcing the importance of tryptophan metabolism in CVD pathophysiology. The findings suggest that manipulating gut microbiota could be a potential therapeutic approach for treating diseases associated with tryptophan metabolism. It is important to note that while these identified biomarkers may have significantly advanced healthcare delivery, their optimal use requires integration with comprehensive clinical data. Their interpretation should always be contextualized within the broader clinical framework, and relying solely on biomarkers without considering the entire clinical picture can lead to incomplete or misleading conclusions.

Future studies should adopt a comprehensive approach that combines retrospective analysis, prospective validation, and the application of artificial intelligence and machine learning techniques to fully establish the predictive value of these biomarkers for cardiovascular diseases.

## Figures and Tables

**Table 1:** Demographic characteristics of participants.

Test	Variable	Control Group (*n* = 112)	CVDs Group (*n* = 112)	*p* Value
General characteristics	Gender (M/F)	68/44	67/45	0.99
	Age	47 (42–51)	53 (46–55)	0.000
	Waist size (cm)	91.5 (85.7–99.2)	98 (91.7–106)	0.000
	Hips size (cm)	104.5 (100–113)	107 (100–115)	0.527
	BMI (kg/m^2^)	29.2 (25.9–33.2)	30.8 (27.5–34.1)	0.029
	Systolic blood pressure (mmHg)	116 (108–123)	126 (119–136)	0.000
	Diastolic blood pressure (mmHg)	75 (69–82)	79 (73–87)	0.006
Glycemic profile	Fasting blood glucose (mmol/L)	5.1 (4.8–5.6)	6.4 (5.4–9.2)	0.000
	HbA1C (%)	5.5 (5.2–5.8)	6.5 (5.9–7.8)	0.000
	C-peptide (ng/mL)	2.4 (1.8–3.2)	3.1 (2.2–4.0)	0.001
	Insulin (uU/mL)	9.7 (6.7–14)	15 (9.9–23.1)	0.000
Lipid profile	Total cholesterol (mmol/L)	5.2 (4.8–5.7)	4.9 (4.2–5.6)	0.014
	HDL-cholesterol (mmol/L)	1.3 (1.1–1.5)	1.1 (0.9–1.4)	0.009
	LDL-cholesterol (mmol/L)	3.2 (2.7–3.9)	3.0 (2.1–3.5)	0.007
	Non-HDL-cholesterol (mmol/L)	3.9 (3.3–4.5)	3.7 (2.9–4.4)	0.182
	Triglyceride (mmol/L)	1.21 (0.9–1.7)	1.68 (1.2–2.4)	0.000
Kidney function	Creatinine (µmol/L)	69.5 (60–81)	74 (64–84.2)	0.020
	Chloride (mmol/L)	69.5 (60–81)	74 (64–84.2)	0.001
	Urea (mmol/L)	101 (100–102)	100 (99–102)	0.004
	Bicarbonate (mmol/L)	4.7 (3.8–5.2)	5 (4.1–6.5)	0.370
	Total protein (g/L)	27 (26–28)	27 (25–28)	0.445
Cardiac function	NT-proBNP (pg/mL)	72.9 (4.1)	73.3 (3.9)	0.057
	Homocysteine (µmol/L)	20.6 (12.6–38.8)	28.7 (15.4–51.1)	0.099
Liver function	Albumin (g/L)	8.7 (7.2–10.2)	9.6 (7.4–11.4)	0.427
	ALT (U/L)	45.1(2.7)	44.8 (3.0)	0.100
	AST (U/L)	21 (15–28)	22 (18–30.2)	0.256
	GGT (U/L)	18 (15–20.2)	18 (15.7–22)	0.001

Data are presented as mean (SD)/median (IQR) for parametric/non-parametric variables. Mean/median between the study cohorts were compared using Student’s *t*/Mann–Whitney U test, respectively. Categorical variables were compared using Fisher’s exact test. Abbreviations: HbA1C, glycated hemoglobin; HDL, high-density lipoprotein; LDL, low-density lipoprotein; NT-proBNP, N-terminal pro–B-type natriuretic peptide; ALT, alanine transaminase; AST, aspartate aminotransferase; GGT, gamma-glutamyl transferase.

**Table 2:** Linear regression analysis to determine the most FDR-significant metabolites associated with CVD, while adjusting for age, gender, BMI, and principal components 1 and 2.

Metabolite	Sub Pathway	Super-Pathway	Estimate	SE	*p*-Value	FDR
Indoleacetylglutamine	Tryptophan Metabolism	Amino Acid	0.836	0.127	4.91 × 10^−10^	4.14 × 10^−7^
Methyl indole-3-acetate	Food Component/Plant	Xenobiotics	0.564	0.093	6.42 × 10^−9^	2.71 × 10^−6^
Indolepropionate	Tryptophan Metabolism	Amino Acid	−0.645	0.132	1.85 × 10^−6^	5.21 × 10^−4^
Indoleacetate	Tryptophan Metabolism	Amino Acid	0.405	0.094	2.32 × 10^−5^	4.89 × 10^−3^

## Data Availability

The datasets used and/or analyzed during the current study are available from the corresponding author upon reasonable request.

## Supplementary Materials

The following supporting information can be downloaded at https://www.mdpi.com/article/10.3390/biom15030377/s1. Table S1. Linear regression analysis to determine metabolites associated with CVD, while adjusting for age, gender, BMI and principle components 1 and 2. Figure S1: Receiver Operating Characteristic (ROC) curve analysis for evaluating the predictive ability of indoleacetylglutamine (and other candidate metabolites) as biomarkers for cardiovascular disease (CVD). The area under the curve (AUC) indicates the discriminatory power of the metabolite(s) in distinguishing between individuals with and without CVD.
