# Supplementary material for: Indoleacetylglutamine Pathway Is a Potential Biomarker for Cardiovascular Diseases
Source: Biomolecules. 2025 Mar 5;15(3):377. doi: 10.3390/biom15030377 (PMC11939839; doi:10.3390/biom15030377)
Supplement: Supplementary file 1 [file biomolecules-15-00377-s001.zip › biomolecules-3463349-supplementary.pdf]

Supplementary Table S1. Linear regression analysis to determine metabolites associated with CVD, while adjusting for age, gender, BMI and principle components 1 and 2.

**Metabolite**	**Sub pathway**	**Super-pathway**	**Estimate**	**SE**	**p-value**	**FDR**
**Indoleacetylglutamine**	Tryptophan Metabolism	Amino Acid	0.836	0.127	4.91×10^{-10}	4.14×10^{-7}
**Methyl indole-3-acetate**	Food Component/Plant	Xenobiotics	0.564	0.093	6.42×10^{-9}	2.71×10^{-6}
**Indolepropionate**	Tryptophan Metabolism	Amino Acid	-0.645	0.132	1.85×10^{-6}	5.21×10^{-4}
**Indoleacetate**	Tryptophan Metabolism	Amino Acid	0.405	0.094	2.32×10^{-5}	4.89×10^{-3}
**N-acetyltyrosine**	Tyrosine Metabolism	Amino Acid	-0.220	0.055	8.35×10^{-5}	1.16×10^{-2}
**2-hydroxyhippurate (Salicylurate)**	Benzoate Metabolism	Xenobiotics	0.920	0.231	9.19×10^{-5}	1.16×10^{-2}
**Glucose**	Glycolysis, Gluconeogenesis, and Pyruvate Metabolism	Carbohydrate	0.185	0.047	9.59×10^{-5}	1.16×10^{-2}
**Pyridoxate**	Vitamin B6 Metabolism	Cofactors and Vitamins	0.434	0.114	1.86×10^{-4}	1.96×10^{-2}
**Pantothenate**	Pantothenate and CoA Metabolism	Cofactors and Vitamins	0.194	0.052	2.70×10^{-4}	2.23×10^{-2}
**Deoxycarnitine**	Carnitine Metabolism	Lipid	-0.104	0.028	3.15×10^{-4}	2.23×10^{-2}
**Linoleoylcholine***	Fatty Acid Metabolism (Acyl Choline)	Lipid	-0.247	0.068	3.29×10^{-4}	2.23×10^{-2}
**Citrulline**	Urea cycle; Arginine and Proline Metabolism	Amino Acid	-0.153	0.042	3.44E-04	2.23E-02
**Tartronate (hydroxymalonate)**	Food Component/Plant	Xenobiotics	-0.271	0.075	3.74E-04	2.25E-02
**Gamma-glutamylcitrulline***	Gamma-glutamyl Amino Acid	Peptide	-0.273	0.078	5.72E-04	3.22E-02
**Glycine**	Glycine, Serine and Threonine Metabolism	Amino Acid	-0.105	0.031	6.75E-04	3.56E-02
**1,5-anhydroglucitol (1,5-AG)**	Glycolysis, Gluconeogenesis, and Pyruvate Metabolism	Carbohydrate	-0.529	0.157	8.87E-04	4.27E-02
**Mannose**	Fructose, Mannose and Galactose Metabolism	Carbohydrate	0.175	0.052	9.62E-04	4.27E-02
**1-palmitoyl-2-arachidonoyl-GPC (16:0/20:4n6)**	Phosphatidylcholine (PC)	Lipid	0.134	0.041	1.18E-03	4.88E-02
**N,N,N-trimethyl-5-aminovalerate**	Lysine Metabolism	Amino Acid	0.165	0.050	1.21E-03	4.88E-02

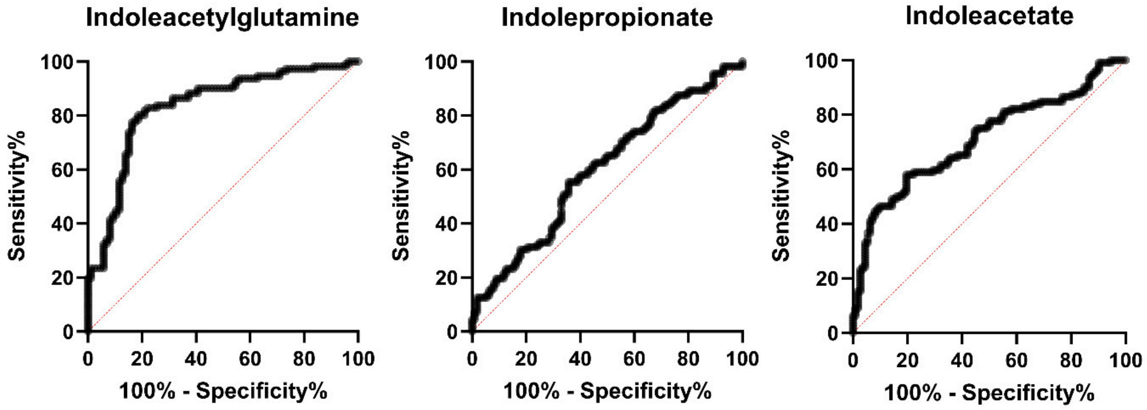

	Area	SE	95% CI	P-value
Indoleacetylglutamine	0.8344	0.029	0.775 – 0.893	<0.0001
Indolepropionate	0.6004	0.037	0.527 – 0.674	0.0094
Indoleacetate	0.7099	0.034	0.642 – 0.778	<0.0001

Supplementary Figure S1: Receiver Operating Characteristic (ROC) curve analysis for evaluating the predictive ability of indoleacetylglutamine (and other candidate metabolites) as biomarkers for cardiovascular disease (CVD). The area under the curve (AUC) indicates the discriminatory power of the metabolite(s) in distinguishing between individuals with and without CVD.
